# Coming to Terms with Complexity: Limits to a Reductionist View of Aging

**DOI:** 10.3389/fgene.2012.00149

**Published:** 2012-08-27

**Authors:** Robert J. Shmookler Reis

**Affiliations:** ^1^Central Arkansas Veterans Healthcare SystemLittle Rock, AR, USA; ^2^Department of Geriatrics, University of Arkansas for Medical SciencesLittle Rock, AR, USA; ^3^Department of Pharmacology/Toxicology, University of Arkansas for Medical SciencesLittle Rock, AR, USA; ^4^Department of Biochemistry and Molecular Biology, University of Arkansas for Medical SciencesLittle Rock, AR, USA

## If Aging is “Not a Process,” then What is It?

My response would be that it is an age-dependent trajectory of interacting system states – the sum of all molecular and physiological states and their interaction networks, many but not all of which shift in a consistent direction over time. This definition broadens our focus to include components that do not themselves depend on age, but which cohabit networks containing components that do. Gene–environment interactions are a case in point, wherein environmental variation can help to shape the age-structure of a population despite being quite obviously independent of age.

Perhaps the best-established genetic pathway to influence lifespan is insulin-like signaling, believed to have evolved at least in part for its ability to maximize reproduction under favorable environments while postponing both reproduction and individual mortality under conditions of crowding or insufficient food (Kenyon, 2005; Kim, 2007; Hanover et al., 2010; Magwire et al., 2010). Since natural populations are polymorphic for ostensibly rate-limiting components of this pathway (Bonafe and Olivieri, 2009), it is likely that individuals genetically predisposed to low insulin-like signaling should survive famine better than those geared for higher signaling and shorter lifespan. This is a conclusion of some import for population biologists, since the age-composition of any population must then be modified by the availability of food. A particularly instructive example is the near-ubiquitous evolutionary requirement for species or their constituent populations to survive extended periods of famine (de Grey, 2005). Groups experiencing more prolonged famines (or just over-wintering, if their lifespans are measured in weeks) will have more diverse age structures, including an increased number of individuals for whom reproduction has been delayed.

The same potential also exists for gene–gene interactions (including genes that dictate dietary preferences) to affect long-term survival. For example, only one component of a gene network may actually be age-dependent, while other genes create the background context of homeostatic states and their oscillations within which age-dependent genes must function. An increased probability of death with age could then arise from components undergoing essentially monotonic age-dependent declines, confronting extreme-value system states (in variable but age-independent parameters) to which they cannot respond adequately in any essential tissue or organ. Alternatively, an age-dependent increase in the variance of system oscillations may exceed the response range of one or more age-independent gene functions. In either case, the precise cause of death or debility will vary in a stochastic way, appearing as the “weakest link” in any one tissue or organism, although the underlying age-associated changes may be common to many or all cell types and individuals (Shmookler Reis, 1989).

## Live Smarter, Live Longer?

There remains, in my view, one last “trivial” explanation for the plateau in mortality, which I believe should be dealt with. I will term it “the perseverance of acquired characteristics,” but I really just mean *learning* in its several forms. If we posit that individuals are to some degree capable, as a function of time, of developing and improving their ability to avoid evitable causes of mortality, then those individuals who managed to survive until late age could *ipso facto* have reduced their late-life risk of death – although aging *per se* might continue unabated. Examples of such learning would include immune memory, strategies to avoid situations and behaviors that place one at increased risk of injury or death, and a reduction in speed of movement or action in recognition of slower response times. My suggestion that immune memory might be involved, in organisms with an adaptive immune system, agrees with the observed age-dependent increase in memory T cells but appears contradicted by the decline with age in recruitment to this niche (Nikolich-Zugich and Rudd, 2010). However, if individuals exist who retain sufficient naïve T cell reserves to augment immune memory at late age, and if those are among the longest-lived in a population, then that subset of the population should see a reduction in their force of mortality. Of course, the strongest evidence for a cessation of aging comes from insects, which lack an adaptive immune system and may be thought incapable of learning. Experimental evidence clearly supports learning by drosophila (Shuai et al., 2011; van Swinderen, 2011), however, and in the rather simple and uniform environments in which they are maintained, the last-surviving individuals might only need to have learned to avoid activities that place them at greatest risk of a collision or loss of balance leading to entrapment on a sticky surface (a major life-hazard for laboratory insects).

## Addressing the Gap between Inbred Model Systems and the Complexity of Natural Populations

It is noteworthy that many gerontologists with broader vision have for some time been “adding back” complexities of genotypic (and more rarely, environmental) variation to aging studies – in keeping with the theoretical advances discussed here, but probably quite independent of them. A recent manifestation of this is the utilization of genetically heterogeneous mouse populations (e.g., four- and eight-way cross progeny; Klebanov et al., 2001; Harrison et al., 2009). Yet another has been the gradual realization that population studies of humans are not only invaluable for initial, weak-inference “discovery” of putative genetic mechanisms underlying diseases and predisposing traits, but are also ultimately needed to validate functional conclusions that arose from experiments with controlled and highly inbred animal populations (Parsons et al., 2005; Szumska et al., 2007).

Human populations of course provide the ultimate in “realism” for both genetic and environmental complexity, but the anticipated harvest of clinically meaningful findings has been delayed and frustrated by the very large numbers of subjects required even for relatively simple traits, and the unforeseen degree of complexity of most quantitative traits (most certainly including longevity and age-dependent diseases) has further diluted the inferential power of such studies (Terwilliger and Weiss, 2003). Nevertheless, with larger and larger cohorts being drafted into studies which interrelate either high-density SNP maps or whole-genome sequencing, with accurate and complete medical and family histories, this type of *post hoc* “experimentation” will soon be pushed to its limits. These studies either have multiple proposed end-points, or else blanket consent forms to permit unforeseeable future applications. Such volumes of data require improved computer algorithms for data analysis, and rigorous statistical evaluation to compensate for multiple-end-point inflation of observed, superficially significant results (Lai et al., 2007; Lam et al., 2009; Erbe et al., 2011).

The worst (and most underappreciated) deficiency of these approaches is the very high level of confounding among the “independent variables” being considered. An obvious example is afforded by genotype-diet interactions, since dietary preferences tend to vary systematically by ethnic group. Another subtle danger that accompanies this particular brave new world is that routine statistical handling of multiple testing (“Bonferroni correction”) can easily be overlooked or ignored. The problem is exacerbated because few investigators (and even fewer reviewers) have the breadth of training to understand both the fundamental biology addressed by a study, and also the arcane “cyber-discipline” of complex-trait analysis along with its own peculiar modes of statistical interpretation, usually trumping model-dependent statistics with permutation analyses. Even among scientists who do appreciate the necessity of multiple end-point compensation, there are many who conveniently forget them when their own data would, if properly adjusted, miss the conventional threshold for significance.

## References

[B1] Bonafe M. Olivieri F. (2009). Genetic polymorphism in long-lived people: cues for the presence of an insulin/IGF-pathway-dependent network affecting human longevity. Mol. Cell. Endocrinol. 299, 118–123 10.1016/j.mce.2008.10.038 19027825

[B2] de Grey A. D. (2005). The unfortunate influence of the weather on the rate of ageing: why human caloric restriction or its emulation may only extend life expectancy by 2–3 years. Gerontology 51, 73–82 10.1159/000082192 15711074

[B3] Erbe M. Ytournel F. Pimentel E. C. Sharifi A. R. Simianer H. (2011). Power and robustness of three whole genome association mapping approaches in selected populations. J. Anim. Breed. Genet. 128, 3–14 10.1111/j.1439-0388.2010.00885.x 21214639

[B4] Hanover J. A. Krause M. W. Love D. C. (2010). The hexosamine signaling pathway: O-GlcNAc cycling in feast or famine. Biochim. Biophys. Acta 1800, 80–95 10.1016/j.bbagen.2009.07.017 19647043 PMC2815088

[B5] Harrison D. E. Strong R. Sharp Z. D. Nelson J. F. Astle C. M. Flurkey K. Nadon N. L. Wilkinson J. E. Frenkel K. Carter C. S. Pahor M. Javors M. A. Fernandez E. Miller R. A. (2009). Rapamycin fed late in life extends lifespan in genetically heterogeneous mice. Nature 460, 392–395 19587680 10.1038/nature08221 PMC2786175

[B6] Kenyon C. (2005). The plasticity of aging: insights from long-lived mutants. Cell 120, 449–460 10.1016/j.cell.2005.02.002 15734678

[B7] Kim S. K. (2007). Common aging pathways in worms, flies, mice, and humans. J. Exp. Biol. 210, 1607–1612 10.1242/jeb.004887 17449826

[B8] Klebanov S. Astle C. M. Roderick T. H. Flurkey K. Archer J. R. Chen J. Harrison D. E. (2001). Maximum life spans in mice are extended by wild strain alleles. Exp. Biol. Med. (Maywood) 226, 854–859 11568309 10.1177/153537020122600908

[B9] Lai C. Q. Parnell L. D. Lyman R. F. Ordovas J. M. Mackay T. F. (2007). Candidate genes affecting *Drosophila* life span identified by integrating microarray gene expression analysis and QTL mapping. Mech. Ageing Dev. 128, 237–249 10.1016/j.mad.2006.12.003 17196240

[B10] Lam A. C. Powell J. Wei W. H. de Koning D. J. Haley C. S. (2009). A combined strategy for quantitative trait loci detection by genome-wide association. BMC Proc 3, S6 10.1186/1753-6561-3-s1-s6 19278545 PMC2654500

[B11] Magwire M. M. Yamamoto A. Carbone M. A. Roshina N. V. Symonenko A. V. Pasyukova E. G. Morozova T. V. Mackay T. F. (2010). Quantitative and molecular genetic analyses of mutations increasing Drosophila life span. PLoS Genet. 6, e1001037 10.1371/journal.pgen.1001037 20686706 PMC2912381

[B12] Nikolich-Zugich J. Rudd B. D. (2010). Immune memory and aging: an infinite or finite resource? Curr. Opin. Immunol. 22, 535–540 10.1016/j.coi.2010.06.011 20674320 PMC2925022

[B13] Parsons C. A. Mroczkowski H. J. McGuigan F. E. Albagha O. M. Manolagas S. Reid D. M. Ralston S. H. Shmookler Reis R. J. (2005). Interspecies synteny mapping identifies a quantitative trait locus for bone mineral density on human chromosome Xp22. Hum. Mol. Genet. 14, 3141–3148 10.1093/hmg/ddi346 16183656

[B14] Shmookler Reis R. J. (1989). Model systems for aging research: syncretic concepts and diversity of mechanisms. Genome 31, 406–412 10.1139/g89-061 2687106

[B15] Shuai Y. Hu Y. Qin H. Campbell R. A. Zhong Y. (2011). Distinct molecular underpinnings of Drosophila olfactory trace conditioning. Proc. Natl. Acad. Sci. U.S.A. 108, 20201–20206 10.1073/pnas.1107489109 22123966 PMC3250181

[B16] Szumska D. Benes H. Kang P. Weinstein R. S. Jilka R. L. Manolagas S. C. Shmookler Reis R. J. (2007). A novel locus on the X chromosome regulates post-maturity bone density changes in mice. Bone 40, 758–766 10.1016/j.bone.2006.10.012 17185055 PMC1861851

[B17] Terwilliger J. D. Weiss K. M. (2003). Confounding, ascertainment bias, and the blind quest for a genetic “fountain of youth”. Ann. Med. 35, 532–544 10.1080/07853890310015181 14649335

[B18] van Swinderen B. (2011). Conditioning to colors: a population assay for visual learning in *Drosophila*. Cold Spring Harb. Protoc. 2011, 1334–1336 10.1101/pdb.prot066522 22046034

